# 고위험 임부를 위한 지지적 프로그램이 불확실성, 불안, 태아 애착에 미치는 효과

**DOI:** 10.4069/kjwhn.2020.06.17

**Published:** 2020-06-23

**Authors:** Hyun Jin Kim, Nami Chun

**Affiliations:** 1 Department of Nursing, Samsung Medical Center, Seoul, Korea; 1 삼성서울병원 간호본부; 2 College of Nursing, Sungshin Women’s University, Seoul, Korea; 2 성신여자대학교 간호대학

**Keywords:** Anxiety, Maternal-fetal relations, Pregnancy, High-risk, Program, Uncertainty, 불안, 모-태아 관계프로그램, 고위험 임신, 불확실성

## Abstract

**Purpose:**

The purpose of this study was to identify the effects of a supportive program on uncertainty, anxiety, and maternal-fetal attachment in high-risk pregnant women.

**Methods:**

The participants were 59 high-risk pregnant women admitted to the maternal-fetal intensive care unit. The control group (n=30) received usual treatment and antenatal care, while the experimental group (n=29) received an additional supportive program. Uncertainty, anxiety, and maternal-fetal attachment were measured in both groups prior to the intervention and at 3 days and 10 days after the intervention (or at discharge). Data were analyzed with the t-test, chi-square test, repeated-measures analysis of covariance, and the Greenhouse-Geisser correction in SPSS version 23.0.

**Results:**

A supportive program including information provision, nutritional care, emotional care, and exercise care was developed from the literature. All variables except women’s length of stay were found to be homogeneous the between experimental and control groups in the pre-test. Length of stay was calculated as a covariate for testing hypotheses. There was a significant difference in state anxiety over time between the two groups, while there were no differences in uncertainty or maternal-fetal attachment.

**Conclusion:**

This supportive program was identified as an effective nursing intervention on state anxiety in high-risk pregnant women during their stay in the maternal-fetal intensive care unit. It is suggested that nurses could apply this program to alleviate high-risk pregnant women’s state anxiety, and that this program could be modified to be more effective on uncertainty and maternal-fetal attachment in high-risk pregnant women.

## Introduction

### 연구 필요성

고위험 임신은 조기 진통, 조기 양막 파수, 자궁경관 무력증, 전치 태반 등의 문제로 일반 임신에 비해 임부와 태아의 건강과 생명에 부정적 영향을 끼칠 수 있는 가능성이 높은 상태를 말한다[1]. 고위험 임신으로 진단받은 임부는 임신 중에 겪는 문제의 위험을 경감시키고 조기에 발견하기 위하여 전문적인 관리와 치료가 가능한 시설에서 입원 치료를 하게 된다. 입원 치료를 하게 된 고위험 임부는 임신 상태에 대한 정보 부족, 임신 결과의 불확실성, 태아 상태 및 안녕을 예측할 수 없는 상황과 정확하지 않은 정보들로 인해 불안해 하며, 이는 결국 고위험 임부의 인지 평가를 방해하여 스트레스를 일으킨다[2,3]. 고위험 임부의 입원으로 인한 스트레스는 신체적 활동의 제한으로 수면장애, 불편감, 운동 부족 등을 유발하며, 정서적으로는 불안과 우울을 경험하게 되어 임신 유지에 부정적으로 작용한다[4,5]. 고위험 임부의 부정적인 정서 상태는 결국 고위험 임부가 태아와의 관계를 부정적으로 인식하게 되어 태아와의 애착 형성에도 영향을 주게 된다[6]. 따라서 입원한 고위험 임부가 경험하는 다양한 문제들을 해결하기 위해 더 정확한 지식과 정보를 제공받는 것이 필요하고, 신체적·정서적 문제를 완화할 수 있는 개별적이고 효과적인 중재가 요구된다[7]. 그러나 실제 임상에서는 임신 유지를 위한 치료에 집중되어 침상 안정과 약물 투여 등 의학적인 처치에만 머무르고 있으며, 고위험 임부를 위한 다양한 중재를 제공하지 못하고 있는 실정이다[7].

현재까지 연구된 고위험 임부에 관한 중재 연구를 보면 이완요법, 음악요법 등의 단일 요법과 정서적 접근이 대부분이다. 이러한 중재는 고위험 임부의 불안과 혈압, 스트레스를 감소시키고, 말초 피부 온도와 산소 포화도가 증가하는 효과는 있었으나[8,9], 교육자와 고위험 임부 간의 즉각적인 피드백 부족, 연구 대상자들이 수동적으로 제공받는 형식의 중재, 정확한 지식 제공 부족, 신체적 건강 문제 해결에는 적용되지 못한 한계점이 있다. 고위험 임부에게 제공하는 정확한 지식과 정보는 고위험 임부의 불확실성을 낮추고 불안을 경감시키며[10], 적절한 영양 및 스트레스 관리와 운동은 침상 안정을 하는 고위험 임부의 신체와 정서에 긍정적인 영향을 줄 수 있다[11]. 따라서 입원한 고위험 임부가 느끼는 신체적 건강 문제, 정보 부족으로 인한 불확실성, 정서적 긴장, 스트레스, 불안 등을 완화하고, 고위험 임부와 태아와의 애착 향상을 위해 임상적 상황을 반영한 지지적인 간호 중재 개발 및 적용이 필요하다.

이에 고위험 임부의 불확실성, 불안, 모-태아 애착 정도를 파악하고, 고위험 임부가 경험하는 정보 부족, 신체적 건강 문제, 정서적 부담감을 완화시키기 위해 정보 제공, 영양 관리, 정서 관리, 운동 관리 등의 요소가 포함된 지지적 프로그램을 개발하고 적용하여 그 효과를 검증하고자 한다.

### 연구 목적

본 연구는 산모 집중치료실(maternal-fetal intensive care unit, MFICU)에 입원한 고위험 임부를 대상으로 지지적 프로그램을 개발하고 효과를 검증하기 위함이며, 구체적 가설은 다음과 같다.

가설 1. 지지적 프로그램을 제공받은 실험군과 대조군 간에는 시간이 경과함에 따라 불확실성 정도에 차이가 있을 것이다.

가설 2. 지지적 프로그램을 제공받은 실험군과 대조군 간에는 시간이 경과함에 따라 불안 정도에 차이가 있을 것이다.

가설 3. 지지적 프로그램을 제공받은 실험군과 대조군 간에는 시간이 경과함에 따라 모-태아 애착 정도에 차이가 있을 것이다.

## Methods

Ethics statement: This study was approved by the Institutional Review Board of Samsung Medical Center (2017-07-060-003). Informed consent was obtained from the participants.

### 연구 설계

본 연구는 일개 의료기관에서 수행됨으로 인해 발생할 수 있는 실험 효과 확산의 가능성을 고려하여 비 동등성 대조군 전후 시차 설계를 이용한 유사 실험연구이다.

### 연구 대상

본 연구의 대상자는 서울 S 종합병원 산모 집중치료실에 입원한 고위험 임부 중 연구 목적을 이해하고 본 연구에 참여하기로 서면 동의한 대상자로 선정하였으며, 대상자 기준은 다음과 같다. 임신 20주부터 33+6주 이내의 1) 조기 진통, 2) 조기 양막 파수, 3) 자궁경부 무력증, 4) 양수 과소, 양수 과다, 5) 자궁 내 발육 지연, 6) 임신성 고혈압, 7) 임신성 당뇨, 8) 전치 태반 등의 진단을 받은 임부였다. 제외 기준은 1) 정신질환을 진단받거나 향정신성 약물을 복용하는 자, 2) 의사소통에 어려움이 있는 자, 3) 의료진 판단 하에 상태가 위중하거나 응급 분만이 예상되는 고위험 임부는 제외하였다. 대상자 수는 양측검정에서 유의수준 .05, 검정력 80%로 하고, 유사한 선행 연구 결과에서 산출된 효과 크기(d=1.23)를 참고하여 큰 효과 크기 0.80을 적용하여 산출하였다[12]. G*power 3.1.9.2 분석 결과 독립표본 t-test에서 이 조건을 충족하는 대상자 수는 26명이었으며 탈락률을 고려하여 실험군과 대조군을 각 30명으로 선정하였다. 연구 진행 과정에서 실험군 1인이 입원 3일째 퇴원하여 최종 분석 대상은 실험군 29명, 대조군 30명이었다. 중재 오염의 위험을 줄이기 위해 대조군 배정을 우선적으로 하여 측정을 완료한 후 실험군을 모집하여 중재를 제공하였다.

### 연구 도구

본 연구에 사용된 모든 도구는 원 개발자와 한국어 번역판 도구 표준화 연구자에게 승인을 받거나 구매하였다.

#### 불확실성

불확실성은 Mishel [13]이 개발한 Mishel Uncertainty in Illness Scale를 영어와 한국어에 능통한 간호학과 교수 1인이 번역본을 완성하고, 이중 언어에 능통한(한국어, 영어) 2인이 영어로 역번역을 하였다. 이 척도는 모호성 13문항, 복잡성 7문항, 비예측성 7문항, 정보 결여 5문항으로 총 33문항의 5점 Likert 척도로 구성되었다. 각 문항은 1점 “전혀 그렇지 않다”에서 5점 “매우 그렇다”까지 측정되며 점수 범위는 최소 33점에서 최대 165점으로 점수가 높을수록 불확실성 정도가 높음을 의미한다. 개발 당시 도구의 신뢰도 Cronbach’s α값은 .70–.91이었고[13] 본 연구에서는 .76이었다.

#### 불안

불안은 Spielberger 등[14]에 의해 개발된 State-Trait Anxiety Inventory를 한국인에 맞게 Han 등[15]이 제작한 State-Trait Anxiety Inventory-form Korean YZ형의 검사 중 상태불안 척도를 사용하였다. 총 20문항 4점 Likert 척도로 각 문항은 1점 “전혀 아니다”에서 4점 “매우 그렇다”까지 측정되며 점수의 범위는 최소 20점에서 최대 80점으로 점수가 높을수록 상태 불안 정도가 높음을 의미한다. 개발 당시 도구의 신뢰도 Cronbach’s α값은 .92였고[14] 본 연구에서는 .95였다.

#### 모-태아 애착

모-태아 애착은 Cranley [16]가 개발한 Maternal Fetal Attachment Scale를 Lee 등[17]이 번역 및 수정 보완한 것을 사용하였다. 이 척도는 자신과 태아의 구별 3문항, 태아와의 상호작용 2문항, 태아의 특성과 의도에 대한 추측 3문항, 자기 제공 5문항, 역할 취득 4문항으로 총 16문항의 5점 Likert 척도로 구성되었다. 각 문항은 1점 “전혀 그렇지 않다”에서 5점 “항상 그렇다”까지 측정되며 점수 범위는 최소 16점에서 최대 80점으로 점수가 높을수록 모-태아 애착이 높음을 의미한다. Cranley [16]의 연구에서 개발 당시 도구의 신뢰도 Cronbach’s α값은 .80이었고, Kim 등[17]의 연구에서는 .90, 본 연구에서는 .88이었다.

### 지지적 프로그램 개발

#### 초안 개발

입원한 고위험 임부를 대상으로 문헌 고찰, 전문가의 자문, 고위험 임부 간호 경험을 바탕으로 한 프로그램의 구성, 중재 내용, 교육 방법, 교육 도구, 시간 배분 등에 관한 입원한 고위험 임부 지지적 프로그램 초안을 구성하였다.

### 지지적 프로그램의 내용타당도 검증

개발된 초안에 대해 전문가에게 자문을 구하여 일부 중재를 수정 보완하였다. 최종 개발된 프로그램의 내용타당도 검증은 간호학 교수 1인, 산부인과 전문의 3인, 산과 병동과 분만장 파트장을 포함한 고위험 임부 간호를 경험한 간호사 3인, 신생아 중환자실 근무 경험이 있는 간호사 1인, 신생아실 간호사 1인, 국제 모유 수유 전문 간호사 1인, 영양사 1인 등 총 11인에게 내용타당도 검증을 의뢰하였다. 내용타당도 검증은 content validity index (CVI) 0.92 이상을 받았다.

#### 지지적 프로그램의 구성과 내용

지지적 프로그램의 내용은 문헌 고찰, 전문가의 자문을 수렴하고, 연구자의 고위험 임부 간호 경험에 근거하여 고위험 임부가 안정감을 느낄 수 있도록 구체적이고 실질적인 정보 제공을 원칙으로 하여 프로그램의 구성과 내용 및 적용 방법을 정하였다(Table 1).

**구성:** 고위험 임부에게 제공되는 지지적 프로그램은 정보 제공, 영양 관리, 정서 관리, 운동 관리의 4가지 영역으로 구성하였다.

**중재 내용:** 임신과 관련된 정확한 지식을 습득한 임부는 자기 관리 능력이 향상되고, 건강한 임신 상태의 유지와 관련된 자신감을 가질 수 있다[18]. 따라서 정보 제공 영역은 고위험 임부에게 산모 집중치료실 소개 및 병실 생활 안내, 고위험 임부 질환 관련 정보, 미숙아와 신생아의 성장과 발달, 캥거루 케어, 아기 돌보는 법 등의 정보를 제공하였다. 모유 수유는 모유 수유하는 방법, 유축기 사용법, 유방 관리, 모유 저장 관리 등의 정보를 제공하였다.

임신 중 다양한 음식을 섭취하는 것은 양수의 맛을 변화시켜 아기가 여러 가지 음식에 쉽게 적응할 수 있도록 도와준다[19]. 또한, 태아의 체중은 심한 영양 부족일 때는 유산, 미숙아, 조산의 가능성이 있고, 과다한 체중 증가는 거대아, 분만 과정의 어려움을 초래한다[19]. 따라서 영양 관리 영역은 침상 안정을 하는 고위험 임부의 영양 관리에 대한 내용을 위주로 자료를 제공하고, 입원과 동시에 영양과 협진을 통한 전문 영양사의 방문과 상담으로 구성하였다.

정서 관리는 임부의 불안 및 스트레스에 긍정적인 효과에 영향을 주며, 통증 감소에도 효과적이다[20]. 따라서 정서 관리 영역은 미술 치료사와의 1:1 상담과 음악 공연을 통한 스트레스 인지 및 정서 관리로 구성되었다. 미술 치료사와 함께 1:1로 미술 치료, 조물 작품 만들기, 그림 그리기를 진행하였고, 재능 기부를 활용한 음악 공연을 통하여 음악 감상을 진행하였다. 태아 일기 작성은 태아 일기장을 제공하고 자유롭게 아기에게 남기고 싶은 말이나 일기를 작성할 수 있도록 격려하였다.

임신 중 운동은 혈액 순환을 돕고 불안과 긴장을 낮추며 마음을 안정되게 하여, 태아의 건강 상태를 향상시키고 임부의 당뇨를 예방하는 등 모든 측면에서 임부 건강에 도움이 된다[21]. 따라서 운동 관리 영역은 입원한 고위험 임부에게 압박 스타킹을 제공하며 목적과 착용법을 설명하고, 침상 운동 동영상을 임부의 휴대전화로 발송하여, 하루 2회(오전, 오후) 이상 침상 운동을 하도록 독려하였다.

**교육 방법:** Tablet personal computer (PC)를 입원과 동시에 제공하며, 개별 PowerPoint (PPT) 슬라이드 교육, 개별 동영상 감상을 진행하였다. 회진 카드 제공 및 수거, 진료과 의사와 간호사의 상담, 국제 모유 수유 간호사, 영양사의 방문 상담과 관리, 미술 치료사와 상담, 음악 공연 감상, 태아 일기 작성 등의 방법을 적용하였다.

#### 지지적 프로그램 적용

##### 정보 제공

프로그램 중재는 담당 간호사나 연구자가 입원 1일째 산모 집중치료실 소개 및 병실 생활 안내를 책자와 tablet PC를 통해 설명하였다. 고위험 임부 질환은 프로그램 진행을 위해 제작하고, 고위험 임부 진단명에 해당하는 내용으로 구성된 maternity school class를 tablet PC를 통해 설명하고 읽도록 하였다. 정보 제공의 효과를 높이기 위해 고위험 임부에게 중요한 내용에 대해 피드백 질문을 하여 이해 정도를 파악하였다.

고위험 임부 질환 및 여러 상황에 대한 궁금증 해결을 위해 입원 시 프로그램을 위해 제작한 회진 카드를 제공하였다. 회진 카드는 고위험 임부가 진료과 회진 전 궁금한 사항을 미리 작성하고, 담당 간호사나 연구자가 질문의 내용을 파악하여 산과 전문의가 질문에 대답하도록 하였다. 회진 카드는 입원 1일째부터 연구 종료 10일째까지 진행되었고, 시간은 10분에서 60분을 제공하였다. 지속적인 정보와 새로운 주제의 정보 제공을 위해 입원 4일째 신생아 및 미숙아 정보 및 모유 수유 정보에 대해 연구자, 연구 보조자, 국제 모유 수유 간호사가 tablet PC의 PPT 자료와 유인물을 이용하여 구두로 설명하고, 시범을 보였다. 또한, 신생아와 미숙아, 모유 수유 관련 궁금증 해결을 위한 회진 카드를 제공하여 질문과 대답으로 진행하였고, 시간은 30분에서 60분을 제공하였다.

##### 영양 관리

영양 관리는 입원일에 담당 의사가 기본 처방에 영양 관리 처방과, 영양과 협진을 의뢰하고, 중재는 입원 2일째에 제공하였다. 입원 2일째에 전문 영양사가 고위험 임부에게 직접 방문하여 1:1로 고위험 임부의 진단명에 따른 영양 상담, 침상 안정 시 필요한 영양 관리에 대해 유인물과 함께 구두로 설명하였다. 영양 관리는 입원 4일째와 6일째에 전문 영양사가 재방문하여 잘 수행하는지, 어려움은 없는지 사후 관리를 하였다.

##### 정서 관리

정서 관리는 입원 2일째를 시작으로 10일째까지 고위험 임부의 컨디션에 따라 시간을 배분하여 시행하였다. 미술 상담은 미술 치료사가 병실에 직접 방문하여 고위험 임부와 1:1로 조물 만들기, 컬러링 그리기, 그림을 통한 상담 등을 진행하였다. 연구자는 중재가 이루어지기 전 미리 고위험 임부에게 하고 싶은 미술 활동을 파악하고 미술 치료사에게 전달하여 미술 치료사가 필요한 재료를 준비할 수 있도록 하였고, 시간은 30분에서 60분을 제공하였다.

음악 감상은 연구자와 담당 간호사가 고위험 임부에게 공연 시간과 공연 내용이 인쇄되어 있는 유인물을 공연 전 미리 제공하고, 음악가들이 병실에 직접 방문하여 악기를 연주하였다. 음악 감상 시에는 본인이 편안한 자세를 취하도록 하고, 고위험 임부의 개인 요구에 따라 병실 커튼을 열거나 닫아서 진행하였다. 연주곡은 태교 클래식과 일반인들에게 익숙한 음악으로 구성되었으며, 공연 횟수는 중재 기간에 적어도 1회, 시간은 30분을 제공하였다.

태아 일기장은 입원 2일째 연구자가 고위험 임부에게 태아 일기장과 필기도구를 제공하였다. 일기장에 태아에 대한 생각, 입원 생활 등의 이야기를 자유롭게 기록하도록 하였으며, 사진을 붙이는 공간도 제공하여 초음파 사진 등을 간직하거나 붙이도록 하였다.

##### 운동 관리

운동 관리는 입원 3일째에 시작하여 연구 종료 시까지 실시하였다. 입원 1일째 압박 스타킹을 제공하고 목적, 착용법 설명과 시범을 보인 후, 입원 2일째 아침부터 착용하고 저녁에 벗도록 하였다. 침상 운동은 연구자와 담당 간호사가 제작한 동영상을 입원 2일째에 임부의 휴대전호로 발송하였다. 입원 3일째부터 연구자나 담당 간호사가 임부의 침상으로 가서 동영상을 보면서 운동 목적과 방법을 시범과 함께 설명하였다. 이후 입원 후 10일까지 하루 2회(오전, 오후) 이상 편안한 시간에 실시하도록 하였다. 운동 유형은 침상 내에서 머리, 목, 손, 팔, 다리 및 발의 관절 운동으로 구성되었으며 총 운동 시간은 20분 07초이다. 운동은 임부의 상태에 따라 모두 또는 일부분을 할 수 있도록 설명하였다.

### 연구 진행 절차

대상자 모집은 산모 집중치료실에 입원한 고위험 임부를 대상으로 연구자가 연구의 목적, 필요성, 방법 등에 대해 설명한 후 자발적으로 참여 의사를 보인 대상자에게 서면 동의서를 받았다. 수집된 자료는 연구목적 이외에 공개되거나 사용되지 않으며, 대상자의 개인정보는 비밀로 유지하되 익명성이 보장됨을 설명하고, 대상자가 원할 경우 언제든지 프로그램 참여를 거부하거나 중단할 수 있음을 설명하였다. 자료 수집은 시험 효과의 확산을 방지하기 위하여 대조군 자료 수집을 종료한 후 실험군 자료 수집을 실시하였다.

장소는 산모 집중치료실 병실, 대상자는 서울 삼성서울병원의 MFICU에 입원한 고위험 임부, 프로그램 적용은 2017년 9월 1일부터 12월 31일까지 진행하였다.

#### 프로그램 운영자 및 전문가 준비

연구자는 산과 병동 간호사로 10년간 근무하였고 신생아 중환자실 근무 경력이 있으며, 국제 모유 수유 전문가로 산모 교육 프로그램을 다수 이수하였으며 대학원에서 여성건강 간호학을 전공하였다. 프로그램 진행을 위해 서울시 S 종합병원의 산과 병동 의료진과 간호사, 영양사, 국제 모유 수유 간호사, 미술 심리 치료사, 음악 단체를 섭외하여 참여하도록 하였다. 사전에 참여자에게 프로그램 내용과 취지, 주의 사항에 대해 직접 설명하였고 프로그램이 일관성 있게 진행되도록 하였다.

#### 사전 조사

**Figure. 1.**
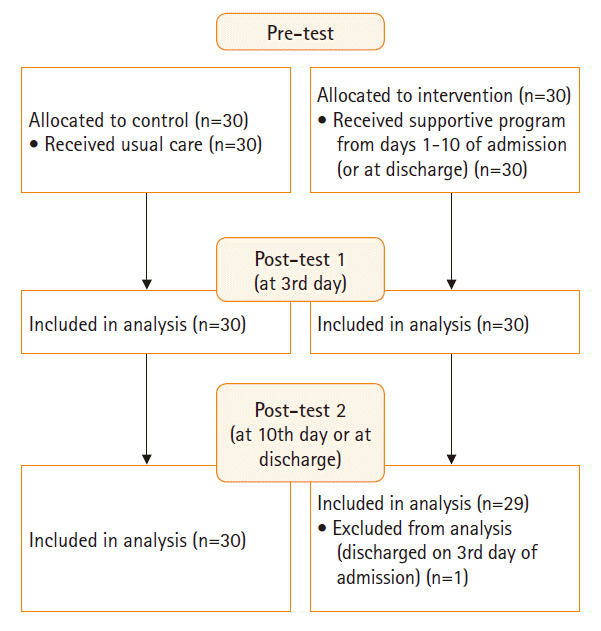
Flow of participants.

사전 조사는 두 군 모두 입원 1일째 실험군과 대조군의 일반적 특성, 산과적 특성, 불확실성, 불안, 모-태아 애착을 조사하였고, 설문지 작성은 15분 정도가 소요되었다(Figure 1).

#### 중재 적용

실험군에게는 평균 재원 일수를 고려하여 입원 1일째부터 10일째까지 본 연구자가 개발한 지지적 프로그램을 제공하였고, 대조군에게는 기존에 시행하던 일상적 입원 생활 안내, 의료진 정규 회진, 치료와 관련된 일반적인 간호 중재를 제공하였다. 두 군 모두에게 자료 수집이 종료된 후에 소정의 감사 선물을 제공하였다.

#### 사후 조사

사후 조사는 두 군 모두 입원 3일째와 10일째 사전 검사와 같은 종속변수를 같은 방법으로 측정하였고, 설문지 작성은 10분 정도가 소요되었다. 사후 조사는 고위험 임부의 입원 기간을 고려하고, 고위험 임부의 증상이 안정되는 시기가 3일[22]과 10일[23]이라는 선행 연구를 토대로 결정하였다. 그러나 실험군 대상자 중 분만으로 인해 MFICU에 10일째까지 있지 못한 대상자는 분만 후 퇴원일에 3번째 설문지를 작성하였다(Figure 1).

### 자료 분석 방법

본 연구의 수집된 자료는 IBM SPSS Statistics ver. 23.0 (IBM Corp., Armonk, NY, USA)을 이용하여 분석하였다. 대상자의 일반적 특성과 산과적 특성은 실수와 백분율, 평균과 표준편차로 분석하였으며, 종속변수의 등분산은 Levene의 등분산 검정, 실험군-대조군 간의 동질성은 t-test, chi-squared test로 검정하였다. 종속변수의 정규성 검정은 Kolmogorov-Smirnov test, 중재 전·후의 실험군-대조군 간 불확실성, 불안, 모-태아 애착 정도는 independent t-test, 시간 경과에 따른 실험군-대조군 간 종속변수의 변화는 실험군-대조군 간 동질성이 확보되지 않은 재원 일수를 공변량으로 하여 repeated measure ANCOVA로 분석하였다. 구형성이 충족되지 않는 경우에는 Greenhouse-Geisser의 epsilon 교정 분석을 하였다.

## Results

### 대상자의 일반적 특성과 연구변수의 사전 동질성 검증

실험군과 대조군의 일반적 특성은 재원 일수를 제외한 모든 변수에서 동질한 것으로 나타났다(Table 2). 종속변수인 불확실성, 불안, 모-태아 애착에 대한 사전 동질성을 검증한 결과 모든 변수에서 동질한 것으로 나타났다(Table 3). 종속변수의 정규성 검정은 Kolmogorov-Smirnov test 결과 불확실성(*p*=.200), 불안(*p*=.200), 모-태아 애착 정도(*p*=.200)의 모든 변수에서 정규성을 충족하였다.

### 고위험 임부를 위한 지지적 프로그램의 효과 검증

지지적 프로그램이 입원 후 시간 경과에 따른 불확실성, 불안, 모-태아 애착 변화에 미치는 효과를 검증하기 위하여 두 군 간에 동질성이 확보되지 않은 재원 일수를 공변량으로 하여 반복 측정 공분산 분석을 수행하였다. Mauchly의 단위행렬 검정 결과 모든 변수에서 구형성이 충족되지 않아(*p*<.05) Greenhouse-Geisser의 epsilon 교정 분석을 하였다. 가설 검증의 결과는 다음과 같다(Table 4).

● 가설 1. 불확실성은 중재 전, 입원 3일, 입원 10일(또는 퇴원일)에 실험군은 97.31±11.58, 96.31±10.10, 96.66±10.04, 대조군은 96.80±8.97, 95.67±7.53, 91.97±10.16으로 두 집단 간에 유의한 차이가 없어(F=0.76, *p*=.440) 가설 1은 기각되었다.

● 가설 2. 불안은 중재 전, 입원 3일, 입원 10일(또는 퇴원일)의 점수가 실험군은 53.48±14.10, 49.34±13.64, 43.72±13.44, 대조군은 47.30±9.37, 43.00±9.09, 42.93±11.02으로 두 집단 간에 유의한 차이가 있어(F=4.08, *p*=.029) 가설 2는 채택되었다.

● 가설 3. 모-태아 애착은 실험군은 중재 전, 입원 3일, 입원 10일(또는 퇴원일)의 점수가 60.45±9.09, 62.34±8.99, 65.69±9.01, 대조군은 62.10±11.28, 64.13±11.65, 65.40±11.36으로 두 집단 간에 유의한 차이가 없어(F=0.90, *p*=.391) 가설 3은 기각되었다.

## Discussion

본 연구는 침상 안정 중인 고위험 임부의 상황을 고려하여 지지적 프로그램을 개발한 후 tablet PC, 휴대전화, 1:1 대면 방문 등의 방법을 활용하여 다학제적인 접근에 의한 중재를 제공했다는 점에서 그 의의가 있다. 가설 검정 결과를 중심으로 다음과 같이 논하고자 한다.

본 연구에서 불안은 실험군이 대조군에 비해 불안이 통계적으로 유의하게 낮아졌다. 이 결과는 조기 진통 임부를 대상으로 이완요법, 음악요법을 적용하여 상태불안 점수가 낮아졌다는 연구 결과[8,9]와 유사하다. 선행 연구는 단일 중재로 이완요법은 1일 2회를 5일 동안 적용, 음악요법은 1일 3회를 4일 동안 적용하였다. 그러나 본 연구에서 음악요법은 10일의 연구 기간 동안 1회 적용했음에도 불구하고 선행 연구와 유사한 결과가 나타났다. 이는, 산전에 병합 중재가 단독 중재에 비해 임부의 불안 증상을 완화하는 효과가 있다는 선행 연구[24]와 유사하게, 본 연구가 미술 치료사와의 1:1 상담 및 미술 활동, 태아 일기 작성 등 2가지 이상의 중재를 적용한 결과라고 생각된다. 미술은 정서를 비언어적인 방식으로 다루는 것으로, 언어적으로 경험하거나 표현하기 어려운 정서와 반응들을 미술 재료나 이미지를 통해 명확하게 표현할 수 있다. 따라서 본 연구의 중재 활동이 고위험 임부들로 하여금 보다 덜 방어적으로 자기 자신을 개방하고 감정을 발산할 수 있도록 하는 기회가 되었다고 생각한다. 또한, 선행 연구들은 연구자의 지도에 따라 간접적인 방식의 중재가 이루어진 반면, 본 프로그램에서는 고위험 임부가 직접 작품 만들기, 그림 그리기, 태아 일기 작성 등의 활동을 한 것이 고위험 임부의 불안을 낮추는 데 유용했다고 생각된다.

본 연구에서 실험군과 대조군의 불확실성에는 유의한 차이가 없는 것으로 나타났다. 이는 고관절 치환술 수술 대기 환자에게 소책자를 이용하여 정보를 제공한 경우 수술 직전 환자의 불확실성이 감소하였고[25], PPT 슬라이드를 이용한 시청각 자료로 정보를 제공했을 때 자궁적출술 환자의 수술 대기 중 불확실성이 감소했다는 연구 결과[26]와 상반된 결과이다. 선행 연구는 수술로 해결되는 단일 질환이었으나 고위험 임부는 임부 자신과 태아 문제까지 포함된 복잡한 질환인 만큼, 정보 제공만으로는 고위험 임부의 불확실성 감소에 영향을 미칠 수 없었던 것으로 생각된다. 또한, 선행 연구에선 실험군에게 한 가지 주제에 대해 집중적인 정보 제공을 하였고[25,26], 본 연구는 여러 가지 주제의 정보를 제공하였다. 이는 고위험 임부들이 지루해 하지 않는 장점은 있으나, 한 가지 주제에 집중하지 못 했을 수 있다. 특히, 선행 연구에서는 수술 환자의 불확실성 감소를 위해 수술 모형을 이용한 실습, 수술실 이동경로 체험 등의 직접 활동을 하였으나[26], 본 연구의 경우 침상 안정만 하는 제한된 활동이 실험군의 불확실성에 유의한 차이를 보이지 않은 것으로 여겨진다. 본 연구에서 통계적으로 유의미한 결과는 아니었지만 입원 10일(또는 퇴원 시)에 오히려 대조군의 불확실성이 실험군에 비해 낮게 나타났다. 추후 추적 결과 실험군이 입원 기간 동안 상태 변화, 응급 분만 등의 상황적 위기가 대조군보다 상대적으로 더 많은 것으로 파악되었다. 이 결과는 고위험 임부의 불확실성을 감소시키기 위해서는 조기 분만, 부정적 임신 결과 등 개인이 처한 상황을 충분히 반영한 중재가 이루어져야 함을 시사한다.

마지막으로 모-태아 애착은 실험군과 대조군 간에 유의한 차이를 보이지 않았다. 본 연구에서는 모유 수유 동영상, 미숙아 교육, 태교 음악 중심의 공연, 태아 일기 작성을 통해 모-태아 애착을 높이고자 하였다. 고위험 임부를 대상으로 모-태아 애착을 측정한 선행 중재 논문은 없지만, 입원한 조기 진통 임부의 모-태아 애착 행위가 정상 임부와 미혼모 대상 임부들보다 높다는 연구 결과[7]로 유추해 볼 때, 본 연구의 결과는 상반된 결과이다. 이는 임부의 정서에 영향을 끼치는 외부 요인 중 하나인 입원으로 인해 다양한 스트레스에 노출되어 임부의 정서 및 모-태아 애착에도 영향을 끼친 것으로 생각된다[6]. 한편, 면회 제한으로 인해 가족이 함께 있지 못하여 가족의 지지를 받을 수 없고, 태아와의 애착을 위해 배우자의 목소리나 발달을 위한 활동을 할 수 없다는 것도 결과에 영향을 끼쳤을 것으로 생각된다. 임신 중 주변 사람들에게서 받는 심리적 지지는 임부의 태아에 대한 행동과 태도에 영향을 높이는 데 효과가 있었던 결과[6,27]는 배우자의 참여 및 가족 지지의 역할을 강조하고 있다. 또한, 조기 진통 임부는 장기간 입원 시 신체적 불편감으로 인해 태아 애착 행위가 오히려 감소[7]하고, 고위험 임부는 입원 기간이 길어질수록 더 높은 스트레스를 받는다[28]는 것을 고려하지 못한 점으로 생각된다. 이상의 논의를 종합해 볼 때, 본 연구의 대상인 고위험 임부를 위한 지지적 프로그램은 고위험 임부의 불안을 감소시키는 데 효과가 있는 것으로 확인되었다. 또한, 본 연구에서 개발한 프로그램은 MFICU에 입원한 고위험 임부를 위한 최초의 지지적 프로그램 개발 및 중재 연구로, 침상 안정만을 하는 고위험 임부에게 적절한 중재를 적용하기 위해 다양한 방법을 적용한 점, 실제 임상 처방에 중재 부분을 접목한 점, 다양한 전문가에 의해 이루어진 중재라는 점에 의의가 있다. 그러나 본 연구에서는 연구 설계 단계에서 실험 결과 측정을 사전, 입원 후 3일째, 입원 후 10일째로 계획한 것과 달리 대상자의 일부가 입원 후 10일 이내에 응급 분만하는 사례가 발생하여 이 경우에 사후 반복 측정을 퇴원 시에 하게 되었다. 이 결과 사전 동질성 검증에서 실험군-대조군 간 재원 일수에 차이가 있어 이를 공변량 처리하여 보완하고자 하였다. 이 결과는 추후 연구에서 고위험 임부의 응급 분만 사례를 고려한 연구 설계의 필요성을 시사한다. 또한, 환자 개인에게 맞는 활동 선택 및 개인별 접근이 아닌 점이나 연구 기간이 상대적으로 짧은 점, 프로그램 중재 장소가 안정감을 줄 수 있는 공간이 아닌 점 등은 본 연구의 한계점이다. 또한 고위험 임부의 요구가 사전에 조사되지 않은 연구라는 것 역시 본 연구의 제한점으로, 다음 프로그램 개발 시에는 고위험 임부의 요구와 의견을 반영한 설문조사가 먼저 이루어져야 할 것이다.

결론적으로, 본 연구에서 개발한 지지적 프로그램은 고위험 임부의 불안을 낮추는 데에 효과가 있음을 알 수 있다. 그러나 본 연구는 일개 종합병원의 MFICU에 입원한 고위험 임부를 대상으로 하였고, 짧은 기간 동안의 자료 수집에 의한 연구 결과이므로 일반화에는 제한이 있다. 따라서 추후 연구에서는 다양한 기관에 입원한 고위험 임부를 대상으로 예상되는 조기 분만이나 바람직하지 않은 출산 결과 등 고위험 임부의 개인적 상황을 고려하여 차별화된 프로그램을 개발하고 적용할 것을 제언한다.

## Figures and Tables

**Table 1.** A supportive program for high-risk pregnant women

Classification (days after hospitalization)	Learning objectives	Lesson contents	Methods	Tools/lecturer	Time (minute)
1. Information provision (1st, 4th)	Understanding the hospital rooms for high-risk pregnant women	Information on hospital rooms in the ward for high-risk pregnant women	1. Providing an educational video and brochure	- Video	10–30
			2. Q&A	- Tablet PC	
				- Brochure/nurse for high-risk pregnant woman	
	Obstetrics: the participant can understand and state her diagnosis, cause, treatment, and prognosis.	1. Definition of high-risk pregnancy	1. Providing materials for the “Maternity school” class	- PowerPoint	30–60
		2. Treatment related to diagnosis: preterm labor, premature rupture of membrane, placenta previa, gestational diabetes, gestational hypertension	2. Providing Q&A cards for doctors’ rounds	- Tablet PC	
				- Brochure/obstetrics specialist	
	Newborn: the participant can understand and state a definition, related diseases, and how to care of premature baby.	1. Definition of premature babies and normal newborn babies	1. Providing materials on premature babies	- PowerPoint	30–60
		2. Introduction to the method of caring for a premature baby	2. Providing Q&A cards for doctors’ rounds	- Tablet PC/pediatrics specialist	
		3. Kangaroo care			
	Breastfeeding:	1. Understanding why breastfeeding is important	1. Providing an educational video & brochure	- Video	30–60
	the participant is trained in breastfeeding skills and knows the advantage of breastfeeding.	2. Understanding how to breastfeed	2. Lecture	- Tablet PC	
			3. Q&A	- Brochure/international breastfeeding certified nurse	
2. Nutritional care (2nd, 4th, 6th)	The participant can understand and state a pregnant woman’s needs for nutritional management.	1. Understanding the importance of nutritional management during pregnancy	1. One-on-one consultations with a nutritionist	- Video	30–60
		2. Introduction of proper caloric intake and essential nutrients	2. Watching educational video	- PowerPoint/nutritionist	
3. Emotional care (2nd to 10th)	Art therapy: the participant can express her feelings and can get emotional relaxation and support.	1. Drawing one’s thoughts about the present state	1. One-on-one consultation with an art therapist	- Sketchbook	30–60
		2. Expressing one’s feelings through consultation	2. Using a coloring book	- Colored pencil	
		3. Reducing stress through color therapy		- Coloring book/art therapist	
	Music therapy: the participant can reduce her stress and can receive emotional relaxation and support.	1. Listening to live classic music	1. Performance of classical music for a small group	- Classical music/talent donation group	60
		2. Explanation of stories about each piece of music: the meaning, background, and composer			
	Fetus diary: the participant is encouraged to improve maternal-fetal attachment and can tell how much she is anticipating the delivery and baby.	1. Looking back on one’s feelings and thoughts about the fetus and oneself in a day	1. Writing a fetus diary	- Diary/nurse in high risk pregnant woman	30–60
		2. Sharing one’s feelings about the delivery and fetus	2. One-on-one consultation		
4. Exercise care (3rd to 10th)	Education on bed exercise; the participant can activate her physical abilities and engage in muscular strengthening before delivery.	1. Understanding the importance of exercise during pregnancy	1. Following the bed exercise video	- Video	30
		2. Introduction to the bed exercise program for high-risk pregnant woman	2. Providing compression stockings	- Compression stockings/nurse for high-risk pregnant women	
		3. Exercise types are divided into neck, arm (hands, wrists, elbows, shoulders), and leg (foot, ankles, knees) exercises.	3. Q&A		
			4. The participants were given exercise types and times adjusted according to their condition.		

PC: Personal computer; Q&A: question and answer.

**Table 2.** Homogeneity test of general and obstetric characteristics between the experimental and control groups (N=59)

Variable	Categories	n (%) or mean±SD		χ^2^ or t	*p*
		Experimental group (n=29)	Control group (n=30)		
Age (year)		33.79±4.40	33.13±3.40	0.65	.521
Gestational period (week)		29.10±2.92	28.90±0.22	0.22	.825
Length of stay (day)		8.31±2.12	9.53±1.17	–2.73	.009
Religion	Yes	16 (55.2)	12 (40.0)	1.36	.243
	No	13 (44.8)	18 (60.0)		
Occupation	Yes	15 (51.7)	20 (66.7)	1.36	.243
	No	14 (48.3)	10 (33.3)		
Need for financial aid	Yes	8 (27.6)	8 (26.7)	0.01	.937
	No	21 (72.4)	22 (73.3)		
Level of education	High school	4 (13.8)	2 (6.7)		.424^†^
	College or higher	25 (86.2)	28 (93.3)		
Birth experience	Yes	9 (31.0)	7 (23.3)	0.44	.506
	No	20 (69.0)	23 (76.7)		
Experience of preterm birth	Yes	7 (24.1)	4 (13.3)	1.14	.287
	No	22 (75.9)	26 (86.7)		
Experience of treatment for preterm birth	Yes	11 (37.9)	14 (46.7)	0.46	.497
	No	18 (62.1)	16 (53.3)		
Regular antenatal screening	Yes	25 (86.2)	27 (90.0)		.706^†^
	No	4 (13.8)	3 (10.0)		
Experience of artificial insemination	Yes	9 (31.0)	5 (16.7)	1.68	.195
					
	No	20 (69.0)	25 (83.3)		
Diagnosis	Preterm labor	7 (24.1)	9 (30.0)		
	PPROM	4 (13.8)	4 (13.3)		
	Short cervix	10 (34.5)	6 (20.0)		
	Placenta previa totalis	2 (6.9)	1 (3.3)		
	Gestational hypertension	3 (10.3)	4 (13.3)		
	Gestational diabetes mellitus	0 (0.0)	1 (3.3)		
	IIOC	0 (0.0)	1 (3.3)		
	Other	3 (10.3)	4 (13.3)		
Experience of childbirth program	Yes	4 (13.8)	7 (23.3)	0.89	.347
	No	25 (86.2)	23 (76.7)		

IICO: Incompetent internal os of cervix; PPROM: Preterm premature rupture of membranes.

† Fisher’s exact test.

**Table 3.** Homogeneity test of research variables in the experimental and control groups (N=59)

Variable	Mean±SD		t	*p*
	Experimental group (n=29)	Control group (n=30)		
Uncertainty	97.31±11.58	96.80±8.97	0.19	.850
Anxiety	53.48±14.10	47.30±9.37	1.98	.054
Maternal-fetal attachment	60.45±9.09	62.10±11.28	–0.62	.539

**Table 4.** Comparison of uncertainty, anxiety, and maternal-fetal attachment between the groups over time (N=59)

Variable	Group	Mean±SD		Source	F	*p*
		Experimental group	Control group			
Uncertainty	Pre-test	97.31±11.58	96.80±8.97	Group	0.02	.890
	Post-test 1	96.31±10.10	95.67±7.53	Time	1.53	.224
	Post-test 2	96.66±10.04	91.97±10.16	G×T	0.76	.440
Anxiety	Pre-test	53.48±14.10	47.30±9.37	Group	1.69	.199
	Post-test 1	49.34±13.64	43.00±9.09	Time	0.61	.504
	Post-test 2	43.72±13.44	42.93±11.02	G×T	4.08	.029
Maternal-fetal attachment	Pre-test	60.45±9.09	62.10±11.28	Group	0.00	.980
	Post-test 1	62.34±8.99	64.13±11.65	Time	0.48	.581
	Post-test 2	65.69±9.01	65.40±11.36	G×T	0.90	.391

Post-test 1: 3 days after admission; post-test 2: 10 days after admission (or at discharge); G×T: group×time. The covariate was length of stay (=8.93) in the analysis of covariance test.
